# Mutation affecting the proximal promoter of *Endoglin* as the origin of hereditary hemorrhagic telangiectasia type 1

**DOI:** 10.1186/s12881-017-0380-0

**Published:** 2017-02-23

**Authors:** Virginia Albiñana, Ma Paz Zafra, Jorge Colau, Roberto Zarrabeitia, Lucia Recio-Poveda, Leticia Olavarrieta, Julián Pérez-Pérez, Luisa M. Botella

**Affiliations:** 1 Centro de Investigaciones Biológicas, Consejo Superior de Investigaciones Científicas (CSIC), and Centro de Investigación Biomédica en Red de Enfermedades Raras (CIBERER), Ramiro de Maeztu 9, Madrid, 28040 Spain; 2 HHT Spanish Unit, Hospital Sierrallana and Centro de InvestigacionBiomedica en Red de Enfermedades Raras (CIBERER), Torrelavega, Santander Spain; 3 Secugen SL, Madrid, Spain

**Keywords:** Hereditary hemorrhagic telangiectasia (HHT), Rare disease, Endoglin promoter, Transcription regulation

## Abstract

**Background:**

Hereditary hemorrhagic telangiectasia (HHT) is a vascular multi-organ system disorder. Its diagnostic criteria include epistaxis, telangiectases in mucocutaneous sites, arteriovenous malformations (AVMs), and familial inheritance. HHT is transmitted as an autosomal dominant condition, caused in 85% of cases by mutations in either Endoglin (*ENG*) or Activin receptor-like kinase (*ACVRL1/ACVRL1/ALK1)* genes. Pathogenic mutations have been described in exons, splice junctions and, in a few cases with ENG mutations, in the proximal promoter, which creates a new ATG start site. However, no mutations affecting transcription regulation have been described to date in HHT, and this type of mutation is rarely identified in the literature on rare diseases.

**Methods:**

Sequencing data from a family with HHT lead to single nucleotide change, c.-58G > A. The functionality and pathogenicity of this change was analyzed by in vitro mutagenesis, quantitative PCR and Gel shift assay. Student *t* test was used for statistical significance.

**Results:**

A single nucleotide change, c.-58G > A, in the proximal *ENG* promoter co-segregated with HHT clinical features in an HHT family. This mutation was present in the proband and in 2 other symptomatic members, whereas 2 asymptomatic relatives did not harbor the mutation. Analysis of RNA from activated monocytes from the probands and the healthy brother revealed reduced ENG mRNA expression in the HHT patient (*p =* 0.005). Site-directed mutagenesis of the ENG promoter resulted in a three-fold decrease in luciferase activity of the mutant c.-58A allele compared to wild type (*p =* 0.005). Finally, gel shift assay identified a DNA-protein specific complex.

**Conclusions:**

The novel *ENG* c.-58G > A substitution in the *ENG* promoter co-segregates with HHT symptoms in a family and appears to affect the transcriptional regulation of the gene, resulting in reduced ENG expression. ENG c.-58G > A may therefore be a pathogenic HHT mutation leading to haploinsufficiency of *Endoglin* and HHT symptoms. To the best of our knowledge, this is the first report of a pathogenic mutation in HHT involving the binding site for a transcription factor in the promoter of *ENG.*

## Background

Hereditary hemorrhagic telangiectasia (HHT), also known as Rendu-Osler-Weber syndrome [1, 2], is a vascular disorder with autosomal dominant inheritance. The latest epidemiological data reveal that HHT affects approximately 1 in every 5,000 individuals [2]. The clinical symptoms, known as the Curaçao criteria [3], diagnose an HHT patient when at least 3 of the 4 criteria are present: epistaxis (nose bleeds), mucocutaneous telangiectases, arteriovenous malformations (AVMs) affecting mostly the lung, liver and brain, and an autosomal dominant pattern of familial inheritance. Hereditary Hemorrhagic Telangiectasia is caused by mutations in at least one of the 3 genes involved in the transforming growth factor-beta (TGF-β) signaling pathway [4]. *Endoglin* (*ENG*, chromosome 9q34), *Activin A receptor type II*-like 1 (*ACVRL1/ACVRL1/ALK1,* chromosome 12q13), and *MADH4/SMAD4* (chromosome 18q21) mutations cause HHT1 (OMIM 187300), HHT2 (OMIM 600376), and the combined Juvenile Polyposis/HHT (JP/HHT) syndrome (OMIM 175050), respectively [5–7]. There are two further unidentified genes that can cause HHT: HHT 3 between 141.9 and 146.4 Mb on chromosome 5q and HHT4 on chromosome 7p between D7S2252 and D7S510.130 [8, 9]. Recently, BMP9 mutations have been shown to give rise to HHT5, although the contribution of BMP9 mutations to HHT is estimated to be very low (<1%) [10].

The genes that are mutated in HHT encode proteins that mediate signaling by the TGF-β superfamily. In the TGF-β signaling cascade, the type II receptor cooperatively recruits and transphosphorylates the type I receptor by direct contact with TβRI [11]. In endothelial cells, upon ligand binding, TβRII can associate with two different TGF-β type I receptors: ALK-5 or ALK-1 [12]. Endoglin is an auxiliary receptor that modulates both complexes in opposite manners. Thus, whereas Endoglin promotes signaling through ALK-1, it inhibits the ALK-5 pathway [13]. In turn, ALK-1 and ALK-5 activate distinct R-Smad pathways, resulting in opposing endothelial cell responses in proliferation, migration, and pro- or anti-angiogenic gene expression.

More than 600 different mutations have been identified in ENG and ACVRL1 in HHT families (HHT mutation database; http://www.arup.utah.edu/database/hht/).

These mutations range from single base-pair changes to large deletions of multiple exons and are of all types, including substitutions, duplications, and deletions. There are no common mutation “hotspots” in either gene, and mutations have been observed across all coding regions.

Considering substitutions, missense mutations are the most common type observed in *ENG* and *ACVRL1*, and mutations have been identified in all exons of both genes, including exon/intron boundaries and splice-site junctions. However, the proportion of mutations causing a frameshift or stop codon in the protein (i.e., indels and non-sense mutations) are more frequent in *ENG* than in A*CVRL1* [14–16].

Although many ENG mutations have been identified in the extracellular region of the protein, which is the largest part of the protein, very few mutations have been identified in the transmembrane and cytoplasmic domains [16]. Large deletions or duplications of one or more exons account for 6–10% of all *ENG* and *ACVRL1* mutations [17]. Large deletions in the *Endoglin* gene, encompassing promoter and several exons or the whole gene, have previously been described by our group [18]. Mutations in the 5′UTR region of ENG (c.-9G > A and c.-127C > T) have also been found to cause HHT. They were determined to be pathogenic by creating an alternative, out-of-frame transcription start codon, but the possibility of altering binding sites for transcriptional factors could not be discarded. Interestingly, (c.-9G > A) is a hypomorphic variant that can be detected in a homozygous state.

These mutations emphasize the need for including the *ENG* 5′UTR region in routine molecular diagnostic testing for HHT [19]. However, to date, few mutations affecting a site in the promoter of either *Endoglin* or *ACVRL1/ALK1* have been reported. A mutation in the regulatory site present in intron 6 (c.772 + 27G > C) of *ACVRL1* has been described in an HHT2 Chinese family [20].

Although the 5′-flanking region of the *Endoglin* gene lacks consensus TATA and CAAT boxes, it contains GC-rich regions and consensus motifs for Sp1, ets, GATA, and TGF-β (Smads), and estrogen-responsive elements. The upstream −400/+341 region is able to display tissue-specific activity in human endothelial cells. Analysis of various deletion constructs demonstrated the existence of a basal promoter region within the −81/+350 fragment [21].

In the present study, we describe, for the first time, a pathogenic mutation that causes HHT type 1 by affecting a single base pair nucleotide change at the c.-58G > A position of the proximal promoter of *Endoglin*, which is embedded in this basal promoter region*.*

## Methods

### Patient samples

Blood samples were collected at different hospitals and sent to our institute. They were received on the day after blood extraction. Informed consent was obtained from each patient. HHT diagnosis was based on the clinical Curaçao criteria [3]. Each sample was anonymously treated and identified with a code number.

### Ethics statement request

The human blood samples and clinical data reported in this manuscript were obtained with prior approval from the appropriate ethics committees of the CSIC for the research conducted in the Centro de Investigaciones Biológicas (CIB, Madrid), the CEIC (Clinical Research Ethics Committee) of the Cantabrian Health Service for patients at the Hospital of Sierrallana (Torrelavega, Santander, Cantabria), and the Medical Genetics department of the Hospital Valdecilla (Santander, Cantabria). Research was carried out in compliance with the Helsinki Declaration (http://www.wma.net/en/30publications/10policies/b3/index.html), keeping the results strictly confidential, with numerical codes for patient identification. http://www.wma.net/en/30publications/10policies/b3/index.html.

### DNA extraction and mutation analysis

Genomic DNA was extracted from peripheral blood, using the *QIAamp* kit (Qiagen, Gmbh, Germany). The coding exons from *Endoglin*, the 9 translated exons from *ACVRLK1/ALK1,* including exon 1 (transcribed but not translated), the exon intron boundaries (50 bp), and the proximal promoters of both genes were amplified by PCR and then sequenced using specific primers. MLPA was also performed for both genes to detect any changes in copy number. The primer sequences and PCR conditions have already been reported [4, 14]. PCR products, which were excised and cleaned from the gel (Millipore, Germany), were sequenced in forward and reverse orientation on an Applied Biosystems sequencer using the dye terminator sequencing kit according to the manufacturer’s instructions. Sequences were compared with the following references for *ACVRL1/ALK1:* OMIM 601284 Gene ID: 94; Genomic ID: NG_009549.1; c-DNA ID: NM_000020.2; and Protein ID: NP_000011. For *ENG*, the references were OMIM 131195 Gene ID: 2022; Genomic ID: NG_009551.1; c-DNA ID: NM_001114753.1; and Protein ID: NP_001108225.12. The following database was used for HHT: http://arup.utah.edu/database/HHT/. Next, if no changes were found in the DNA sequence comparisons, MLPA [22] was performed according to the manufacturer’s instructions using the P093 Salsa MLPA HHT/PPH1 probe set (MRC-Holland, Amsterdam, The Netherlands). DNA sequences and MLPA analyses were performed by Secugen S.L (Madrid, Spain). In the index case analyzed in this manuscript, only a change G > A, in position −58 bp of *Endoglin* promoter was observed; it was considered a variation of the gene and had an unknown effect (VUS).

### Site-directed mutagenesis

Site-directed mutagenesis was carried out from a construct encompassing 350 bp upstream of the transcription initiation start of *ENG,* including a 150-bp transcribed untranslated region before the codon start with the luciferase reporter gene in the pXP2 vector (−350/+150 *ENG* pXP2). Mutagenesis was performed using the Quick Change Site-Directed Mutagenesis kit from Agilent Technologies, according to the manufacturer’s instructions. These procedures were followed by DpnI digestion to eliminate the original wild type DNA template. Oligonucleotides used for the mutagenesis were: *ENG* -58Mut:

*Fwd*: 5′- CCACAGCCCTGCCACTGGACA-3′

*Rev*: 5′-TGTCCAGTGGCAGGGCTGTGG-3′

### Cell culture

Human microvasculature endothelial cells, HMEC-1, were grown on 0.2% gelatin (Sigma-Aldrich) in PBS-coated plates in MCDB-131 medium (Gibco) supplemented with 10% FCS (Gibco), 1 ng/ml of EGF and 1 μg/ml of hydrocortisone (Sigma), 2 mM L-glutamine (Gibco) and 100 U/ml of penicillin and streptomycin (Gibco).

### Transfections and luciferase assays

Superfect® Transfection Reagent (Qiagen), was used for transient transfection according to the manufacturer’s instructions. A total of 1 μg from Luciferase Plasmid Reporters DNA [either wild type or mutated (−350/+150 ENG pXP2)] and 20 ng of pSV40-βgal reporter plasmid (for transfection normalization) were incubated in Opti-MEM (Gibco) with Superfect for 15 min at room temperature. The DNA-transfection reagent complexes were added to cells that were 80% confluent in P-24 wells. Transfection was allowed to proceed for 24 h at 37 °C and 5% CO_2_.

Cells were lysed in 1× lysis buffer from Promega. Relative units of luciferase (Promega luciferase reagent) were measured in a Glomax luminometer (Promega), and β-galactosidase units as measured by Galacto-light (Tropix) were used to normalize transfection efficiency. Each condition was repeated in triplicate. Treatments with TGF-β1 (Preprotech) proceeded for 24 h with 10 ng/ml. The control reporters of TGF-β, CAGA-luc, and BRE-luc were used in parallel as reporter controls of the TGF-β response.

### RNA gene expression

#### Isolation of MNCs, culture and RNA extraction

From 5 ml samples of peripheral blood from patients, the total fraction of MNCs (mononuclear cells) was isolated in a Ficoll gradient (Lymphocyte Separation Medium, Lonza). The MNC fraction was plated and cultured for 24 h in DMEM medium containing 10% fetal bovine serum (FBS, Gibco). Total RNA from these activated MNCs was isolated using the NucleoSpin RNA purification kit from Macherey-Nagel (GmbH&Co).

#### Quantitative RT-PCR

For quantitative analysis of ACVRL1/ALK1 and ENG transcripts, total RNA was reverse-transcribed using the Reverse Transcriptase AMV cDNA Synthesis kit (Roche). The resulting cDNA was used as a template for real-time PCR performed using the SYBR Green PCR system (BioRad), with the following primers. *ACVRL1/ALK1: Fwd* 5′-ATCTGAGCAGGGCGACAGC-3′ and Rev 5′-ACTCCCTGTGGTGCAGTCA-3′; ENG Fwd 5′-GCCCCGAGAGGTGCTTCT-3′ and Rev 5′-TGCAGGAAGACACTGCTGTTTAC-3′. As an internal control, mRNA levels of 18S were measured using these primers: Fwd 5′-CTCAACACGGGAAACCTCAC-3′ and Rev 5′-CGCTCCACCAACTAAGAACG-3′. Amplicons were detected using an iQ5 system (BioRad). Each experiment was performed in triplicates.

### Gel shift assay (EMSA) of proximal *ENG* mobility

Nuclear extracts from confluent HMEC-1 cultures were obtained using a Nuclear Extract kit (Active Motif). The amount of protein was determined by the Bradford technique (Bio-Rad), and 10 μg of total nuclear extract was used for incubation with the 5′ biotinylated oligonucleotide probe from Sigma Aldrich. Complementary oligonucleotides encompassing the −69 to −56 region of *Endoglin* promoter were annealed by heating 95 °C for 3 min, slowly cooling down to RT, and then incubating overnight at 4 °C.

**Table:** 

Biotinylated wt fwd −69/-56	5′- GGTGCCCGCCCGCAGCCCTGCCAC -3′
Biotinylated wt rev −69/-56	5′- GTGGCAGGGCTGCGGGCGGGCACC -3′

The gel shift assay was carried out following the protocol of Gelshift™ Chemiluminescent EMSA. Briefly, 20 ng annealed probe was incubated with 10 μg nuclear extract in the presence of 2 μg of polydI-dC as non-specific competitor. Specific competition assays occurred with an excess (100-fold) of wt cold unlabeled annealed probe and the same amount of mutated cold unlabeled probe. Protein-DNA binding was allowed to proceed for 30 min at 4 °C; then, samples with loading buffer were loaded in a 6% non-denaturing polyacrylamide gel in 0.5× TBE and electrophoresed for 1 h at 4 °C and 150 V. Next, the DNA-protein complexes and free probe were transferred to a nylon membrane by electroblotting. DNA-protein complexes were crosslinked by UV to the nylon membrane. The membrane was processed by blocking and was then incubated with the streptavidin-HRP conjugate for 30 min. After being washed, the membrane was developed by the Super signal chemiluminescent Kit (Thermo-Scientific), according to the manufacturer instructions, and visualized with a Molecular Imager, Chemi-Doc.

### Statistics

Data were subject to statistical analysis, and the results are shown as the mean ± SD. Differences in mean values were analyzed using Student’s *t*-test. In the figures, the statistically significant values are marked with asterisks (*p < 0.05; **p < 0.01; and ***p < 0.005).

## Results

### Correlation between the presence of deletions in ENG and clinical phenotype

DNA from a patient presenting epistaxis, gastric bleeding, telangiectasia and liver arteriovenous malformations was referred to our laboratory for genetic diagnosis after a clinical diagnosis of HHT was presented. After sequencing all exons, intron boundaries, and proximal promoter regions of *ACVRL1/ALK1* and *ENG* and the MLPA of both genes, we could not find any mutations in the DNA. Only a change, G > A, in position −58 bp of *Endoglin* promoter was observed, and it was considered to be a variation of the gene with an unknown effect.

**Fig. 1:**
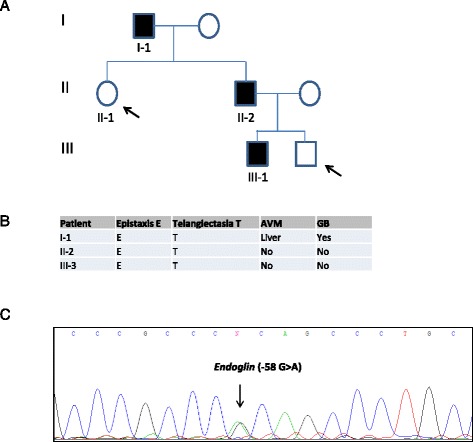
**a** Schematic representation of the family tree with the distribution of HHT and normal relatives. **b** Clinical diagnostic criteria of the affected relatives. **c** Mutated sequence corresponding to this particular HHT family, showing the nucleotide substitution c.-58 G > A at the proximal promoter of *Endoglin.* Arrows point to the two unaffected individuals, who were also sequenced

To determine whether the change could be the mutation causing HHT symptoms, 4 direct relatives of the index case were sequenced. The c.-58 G > A change was identified in two of them, and those two presented epistaxis and telangiectasia, whereas the other two relatives had no apparent clinical symptoms (epistaxis or telangiectasia in either adult). The phenotype-genotype correlation between led us to consider that the change could be pathogenic (Fig. 1). Clinically, the index case for HHT (I-1) (Fig. 1) was 79 years old and suffered from frequent epistaxis, exhibited many telangiectases on the face and hands and had arteriovenous malformations in the liver and gastric bleeding; therefore, it could not be considered a “mild” phenotype. Patient II-2 (Fig. 1) was a 45-year-old male who suffered from frequent epistaxis and had several telangiectases on the face and mucosa. No screening for AVMs occurred. The III-3 proband was 19 years old and had mild epistaxis and few telangiectases on the lips and fingertips.

### Site-directed mutagenesis of Endoglin proximal promoter and transfection

To assess whether an *Endoglin* c.-58 bp G > A change could affect endoglin transcription, we performed site-directed mutagenesis on an *Endoglin* promoter pCD105 (−450/+350) with a pXP2 luciferase reporter [23].

HMEC-1 cells were transfected with either the wild type or mutated sequence of the endoglin promoter. To control cell transfection, the pSV40-βgal reporter plasmid was also co-transfected. At the same time, HMEC-1 cells were incubated in the presence or absence of 10 ng/ml of TGF-β1 for 24 h.

**Fig. 2:**
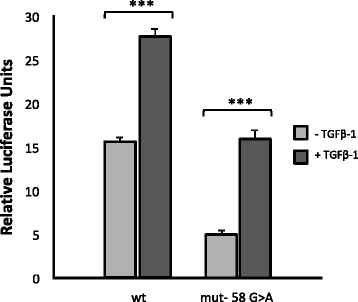
Histograms representing the relative luciferase activity of wild type and mutated *Endoglin* promoter reporters after transfection into endothelial HMEC-1 cells. Site-directed mutagenesis was performed on the wild type promoter/reporter plasmid (350/+350 CD105 pXP2) of Endoglin. The effect of TGF-β treatment is also shown

As shown in Fig. 2, the wild type endoglin promoter had 3-fold more luciferase activity than did the mutated promoter (c.-58 G > A). This difference was statistically significant, *p <* 0.005. TGF-β treatment upregulated both the wild type and mutated promoters; therefore, the mutation does not seem to involve a TGF-β responsive element.

However, there was significantly more relative upregulation (*p <* 0.05) in the wild type than in the mutant after TGF-β treatment.

### RNA endoglin expression of HHT and wild type monocytes activated in vitro

Monocytes do not express significant levels of *Endoglin* [24]. However, the expression of *Endoglin* is triggered during the process of monocyte activation and transition to macrophage.

**Fig. 3:**
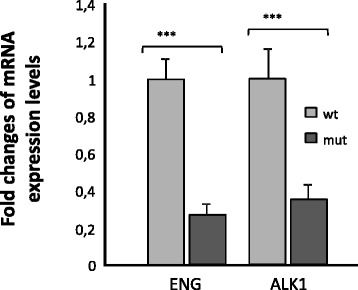
Results of RT quantitative PCR showing the RNA transcription levels of *Endoglin* and *ACVRL1/ALK1* genes of activated monocytes from a HHT patient compared with his control brother. The results are compared to the endogenous control 18S. The qPCR was repeated three times, and the results shown are representative, with triplicates for each sample

Because haploinsufficiency is the mechanism underlying HHT [2], and the (c.-58 G > A) in *Endoglin* promoter seemed to reduce, at least in vitro, the promoter activity in a reporter (Fig. 2), we decided to assess activated monocytes from 1 HHT patient and his healthy brother, evaluating *Endoglin* expression in both patients side by side. In the case of the Endoglin RNA from the HHT patient, activated monocytes expressed significantly less Endoglin and *ACVRL1/ALK1* compared to the healthy donor (*p <* 0.005) (Fig. 3). Figure 3 shows the relative expression of ENG and *ACVRL1/ALK1* of the affected patient compared to the healthy sibling in activated monocytes. The amount of *ENG* RNA in the wild type sample was significantly higher than that in the mutant one (Fig. 3). *ACVRL1/ALK1* expression was also decreased, which may be explained by a crosstalk between both genes, published before [24]. Therefore, these results represent in vivo confirmation of the deficient expression of Endoglin in the HHT patient related with the change (c.-58 G > A) in the promoter. Next, we decided to test whether the binding of a transcription factor could be prevented by changing G to A.

### Changing G > A in the promoter interferes with transcription factor binding

**Fig. 4:**
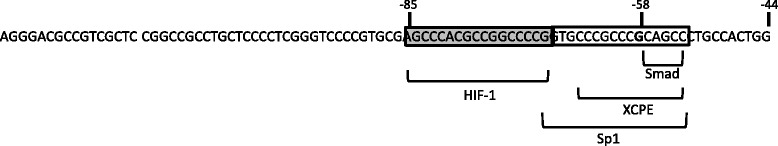
Schematic representation of the proximal promoter region encompassing the −58 (G > A) site and the transcription factors binding to it, according to the MatInspector program (Genomatix)

The MatInspector program (Genomatix) (2005) [25] predicted that the change (−58 G > A) was in a Sp1/GC factor consensus that also overlapped also with SBE (Smad binding elements) and partially overlapped a core region for the core promoter element for RNA pol II (CPE) transcription for TATA-less promoters, in the case of Endoglin. The site where the mutation was placed and the putative binding places for these transcription factors is depicted in Fig. 4.

**Fig. 5:**
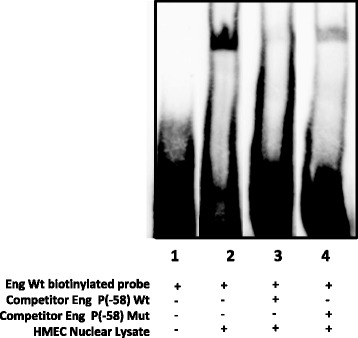
Gel shift assay of nuclear extract from endothelial cells showing a retarded band of protein-DNA, within the proximal promoter of *Endoglin*. Nuclear extract from endothelial cells (HMEC-1 cell line) was incubated with the double stranded biotinylated oligonucleotide, and in the presence of unspecific competitor (poli dI-dC), a retarded band could be detected (lane 2). However, the sample was specifically treated with 100× excess of the unlabeled double-stranded nucleotide (cold probe) (lane 3); when the mutated oligonucleotide was added, the binding efficiency of the probe was not diminished (lane 4). The experiment was repeated 4 times. This image is representative of the obtained results

The next step was to synthesize a double-stranded oligonucleotide probe covering the region encompassing the mutation and then test whether we could detect DNA-protein complexes in a gel shift assay. As shown in Fig. 5, when the nuclear extract from endothelial cells (HMEC-1 cell line) was incubated with the double-stranded biotinylated oligonucleotide, in the presence of unspecific competitor poli (dI-dC), a retardation band could be detected (lane 2). However, the band was not visualized after the sample was incubated with a 100× excess of the unlabeled double-stranded nucleotide (cold probe) (lane 3). When there was competition between the mutated oligonucleotide and the wild type nucleotide, the retarded band was not so efficiently decreased (lane 4). Therefore, there was a protein complex binding the region in which the mutation was placed, which, according to the predictions in Fig. 4, is a complex of the Sp1/GC-rich family of transcription factors. Therefore, the results suggest that the mutation (c.-58 G > A) in the proximal promoter of *Endoglin* precludes or disturbs Sp1 binding to initiate transcription. This explains why transcription is inefficient and Endoglin haploinsufficiency occurs.

## Discussion

HHT is caused by mutations in the transforming growth factor-beta (TGF-β) signaling pathway genes *ENG*, *ACVRL1/ALK1*, and *SMAD4* [5–7], and the more recently identified *BMP9* [9].

Haploinsufficiency has been postulated to be the mechanism of pathogenesis leading to HHT. In this particular case, mutation (c.-58 G > A) in the promoter of *Endoglin* precluded Sp1 binding and the initialization of efficient transcription. Transcription proceeds normally only from the wild type allele. Therefore, the mutation described outlines a new way to generate *Endoglin* haploinsufficiency.

With the advent of next generation sequencing (NGS), a new era has begun in which HHT molecular diagnostics can be performed more quickly, easily, and inexpensively. McDonald and coworkers [15] have set up multi-gene NGS panels for the rapid molecular diagnosis of HHT.

However, approximately 5-10% of individuals clinically identified as having HHT, according to the clinical *Curaçao* criteria, currently have no known genetic cause [15]. Should we look for additional causative genes or genetic modifiers beyond those already described? Alternatively, are deep intronic variants or changes in the regulatory (promoter, enhancer) regions of the already known *ENG, ACVRL1/ALK1, MADH4,* and *BMP9* genes responsible for HHT, which accounts for the percentage of genetically undiagnosed patients?

Current clinical testing protocols, including whole exome sequencing (WES), only involve exons and intronic boundaries. To cover regulatory and deep intronic regions, the advent of targeted whole gene capture of the relevant loci, including introns and UTRs, would be desirable in this scenario. This issue was supported by Marchuk, and the results of the Arup group can serve as an alternative to looking for new HHT genes [26].

Van Sant-Webb and colleagues demonstrated that some HHT patients with no known genetic cause may have a deleterious non-coding region variant in a known HHT gene [27].

One could imagine that including longer upstream regions of the promoters (primarily *ENG* and *ACVRL1/ALK1*) and sequencing the introns would increase the feasibility to find variants affecting the splicing and regulation of the candidate genes, and the rate of genetic diagnosis would increase significantly. The importance of sequencing the entire *ENG* and *ACVRL1/ALK1* (even *SMAD4 or BMP9*) genes, depending on the clinical symptoms, is clear before looking for additional genes.

Thus, the results shown in the present paper constitute an example of the last statement.

Although there have been 2 previous reports of 3 variants affecting the 5′UTR region of ENG of *Endoglin*, two were pathogenic as a result of the generation of an alternative transcription start codon that was out of frame, without excluding transcription factor binding [19]. This is the first work showing that a change in the proximal promoter of *Endoglin* precludes Sp1 transcription factor binding around the −58 bp position in the minus strand. Sp1 was described as an essential factor for *Endoglin* basal transcription [23], in the absence of TATA box.

Altogether, this paper concludes that whole gene sequencing, not just coding regions, of *ENG, ACVRL1/ALK1,* and depending on the phenotype, even of *Smad4* and *BMP9*, may reveal that non-coding regions play a larger role in HHT disease pathogenesis, which may help to cover the full range of genetic diagnoses in clinically diagnosed patients.

## Conclusion

This manuscript constitutes the first work, to the best of our knowledge, demonstrating a mutation in the proximal promoter of *Endoglin* that affects the transcriptional regulation of Endoglin, downregulating it and leading to likely HHT pathogenic manifestations. The change of a single nucleotide precludes the binding of Sp1, which is an essential factor for Endoglin basal transcription, around the −58 bp location in the minus strand.

Many HHT patients with unknown genetic profiles may have mutations located in the regulatory regions of either *Endoglin* or *ACVRL1/ALK1*. This work emphasizes the need for increased coverage by sequencing of non-coding regions in the relevant loci, uncovering regions that have not been observed in exome sequencing.

It is very likely that in HHT we do not need to look for more genes but should instead consider the entire genomic sequence of *ACVRL1/ACVRL1/ALK1* and *ENG* to identify changes in non-coding regions that may affect transcription, splicing and even translation.

## Abbreviations

ACVRL1/ACVRL1/ALK1: Activin Receptor Kinase Like type 1
ALK5: Activin Like kinase Type 5
AVM: Arterio-Venous Malformations
GDF: Growth and Differentiation Factors
HHT: Hereditary Hemorrhagic Telangiectasia
HMEC-1: Human Microvasculature Endothelial Cells
JP: Juvenile Polyposis
MADH4/SMAD4: Mothers Against Decapentaplegic
MLPA: Multiplex Ligation PCR Assisted
R-Smads: Receptor Smads
Sp1: Specific protein 1
TGF-β: Transforming growth factor beta
TβRI/RII: Receptors type I and II of Transforming Growth Factor beta
UTR: Untranslated region
